# Effect of Electrolytic Medium on the Electrochemical Reduction of Graphene Oxide on Si(111) as Probed by XPS

**DOI:** 10.3390/nano12010043

**Published:** 2021-12-23

**Authors:** Andrea G. Marrani, Alessandro Motta, Francesco Amato, Ricardo Schrebler, Robertino Zanoni, Enrique A. Dalchiele

**Affiliations:** 1 Dipartimento di Chimica, Università di Roma La Sapienza, p.le A. Moro 5, I-00185 Rome, Italy; francesco.amato@uniroma1.it; 2 Dipartimento di Chimica, Università di Roma La Sapienza and Instm UdR, Roma p.le A. Moro 5, I-00185 Rome, Italy; alessandro.motta@uniroma1.it; 3 Instituto de Química, Facultad de Ciencias, Pontificia Universidad Católica de Valparaíso, Av. Brasil, Valparaiso 2950, Chile; ricardo.schrebler@pucv.cl; 4 Instituto de Física, Facultad de Ingeniería, Universidad de la República, Julio Herrera y Reissig 565, C.C. 30, Montevideo 11000, Uruguay; dalchiel@fing.edu.uy

**Keywords:** graphene oxide, graphene, electrochemical reduction, non-aqueous electrolyte, silicon surface, cyclic voltammetry, XPS, Raman spectroscopy

## Abstract

The wafer-scale integration of graphene is of great importance in view of its numerous applications proposed or underway. A good graphene–silicon interface requires the fine control of several parameters and may turn into a high-cost material, suitable for the most advanced applications. Procedures that can be of great use for a wide range of applications are already available, but others are to be found, in order to modulate the offer of different types of materials, at different levels of sophistication and use. We have been exploring different electrochemical approaches over the last 5 years, starting from graphene oxide and resulting in graphene deposited on silicon-oriented surfaces, with the aim of understanding the reactions leading to the re-establishment of the graphene network. Here, we report how a proper choice of both the chemical environment and electrochemical conditions can lead to a more controlled and tunable graphene–Si(111) interface. This can also lead to a deeper understanding of the electrochemical reactions involved in the evolution of graphene oxide to graphene under electrochemical reduction. Results from XPS, the most suitable tool to follow the presence and fate of functional groups at the graphene surface, are reported, together with electrochemical and Raman findings.

## 1. Introduction

Graphene, a two-dimensional (2D), single-layer planar sheet of *sp*^2^-hybridized carbon atoms densely packed in a honeycomb crystal lattice, is considered the first true 2D-material (i.e., one atom thick) to become a keystone in material science research ever since it was isolated from lumps of graphite in 2004 by Geim and Novoselov et al. [1,2,3,4,5,6,7]. This success was motivated by its unique electronic, electrochemical, optical, thermal and mechanical properties, leading to its application in a variety of research areas including electronics, sensing, energy conversion and storage, water purification and biomedical applications [1,2,3,5,6,7,8]. Graphene is the first example belonging to the large graphene family of nanomaterials, differing from each other in terms of their number of layers, size and surface properties [1,2,3,6,7]. Among them, graphene oxide (GO) and reduced GO (rGO) are considered the most important derivatives of graphene [6]. Graphene oxide consists of a monolayer of carbon atoms having both (significantly) *sp*^2^-hybridized carbon atoms and (partially) sp^3^-hybridized carbon atoms bearing polar oxygen-containing functional groups (OFGs) distributed on its basal plane (hydroxyl and epoxy) and along its edges (carbonyl and carboxyl) [6,9,10,11,12,13,14]. The type and degree of the functionalization of the OFGs in graphene oxide are variable, principally depending on the different synthetic procedures used [9].

GO has been historically recognized as a precursor to the production of graphene-like materials [9,15,16]. In fact, it can be reduced (or partly reduced) to graphene-like sheets by removing the OFGs [17]. The rGO sheets are typically considered to be a material with intermediate properties compared to GO and pristine graphene [17,18,19], whereas fully reduced GO has properties very close to graphene [9]. In recent years, due to its unique properties such as its hydrophilic character, tunable optical band gap and high chemical reactivity, GO has received much attention for its technological implementation [15,19,20,21]. In fact, GO has been applied in electronic gate dielectric materials, such as thin film transistors and resistive random-access memory, solar cells and biosensors. It was also reported to be a promising material in dentistry and tissue regeneration, as a biomaterial for interfacing cell imaging and drug delivery, and as an efficient cargo platform for cancer theranostics, phototherapy and bioimaging [22,23,24,25,26,27,28]. On the one hand, GO tackles some of the inherent disadvantages of pristine graphene (i.e., its high hydrophobicity, chemical inertness and lack of dispersibility in water), and on the other hand it exhibits some drawbacks (i.e., a low conductivity and amorphous structure) that hinder its broader applications. Hence, in order to benefit from both GO and graphene, a balance between their desirable properties is necessary [20]. Recently, a precise modification of oxygen functionalities on graphene and the graphene oxide basal plane was proposed, in order to finely tune its properties [9,11,29,30,31,32]. The main challenge is to carry out a precise reduction process (using different reducing agents or reduction conditions), with the possibility of controlling both the reduction degree (the OFGs’ content) and the selectivity of the key OFGs’ removal [11,33]. Moreover, when dealing with applications in different optoelectronic and energy conversion and electrochemical storage areas the other very important issue is that the reduction process must be compatible with on-chip integration [9,34,35,36,37].

The reduction of GO layers can be achieved using thermal, chemical, photoreduction, microwave irradiation and electrochemical methods [6,9,32,38]. Electrochemical reduction, a room temperature process, is considered a green route, since neither toxic chemicals (which are harmful to both humans and the environment, and can contaminate the final product) nor high temperatures are employed during the electrochemical reduction process [10,16,21,29,39]. Moreover, the simple manipulation of the electrochemical reduction process of the graphene oxide can be achieved through controlling the potential, reduction time and electrolyte choice [10,33,40,41]. Good quality electrochemically reduced GO (erGO) layers can be formed directly onto different substrates such as silicon and indium tin oxide (ITO)/polyethylene terephthalate (PET) flexible substrates with a high interest in conventional optoelectronic devices [42,43,44] and transparent and flexible electrodes in transparent optoelectronic devices with novel functionalities (e.g., flexibility, semi or full transparency [45,46]), respectively [40,47,48]. Despite these practical advantages, the electrochemical reduction of GO is based on a reaction mechanism that has rarely been addressed in the literature, and its elucidation is still a matter of debate [49,50].

In this work, we report a comparative study of the effects of exploiting two different, yet common, electrolyte media in the electrochemical reduction of GO layers deposited on crystalline silicon. Such electrolytes are aqueous and aprotic organic, and are expected to influence the reduction degree and selectivity, as well as the structural properties of the resulting reduced GO layers. These features are investigated in detail by applying a mixed electrochemical/surface science approach based on X-ray photoelectron spectroscopy (XPS), Raman spectroscopy and electrochemical methods, with the aims of helping the assessment of the most opportune conditions for obtaining a chemically controlled high-quality erGO material and increasing the knowledge on the interpretation of the GO reduction reaction path.

## 2. Materials and Methods

All the chemicals used were of analytical reagent grade and used as received. A 4 mg mL^−1^ water dispersion of GO was purchased from Graphenea (Graphenea Headquarters, San Sebastián, Spain), consisting of single layered graphene oxide synthesized with a modified Hummers protocol as indicated by the supplier. This dispersion was then diluted to a 0.06 mg mL^−1^ concentration with distilled water for the following steps to prepare the samples.

Single-sided polished Si(111) wafers (Sieger Consulting), 380 µm thick, n-type (0.005 ÷ 0.02 Ω cm resistivity), with approximate area of 1 cm^2^, were cleaned in a series of organic solvents with different polarity and then placed directly into 40% NH_4_F(aq) (Fluka) for 10 min to etch the native oxide layer and produce a H-terminated Si(111) surface, followed by water rinsing and drying under a stream of N_2_(g).

The formation of GO deposits on silicon was attained by drop-casting 50 µL of a 0.06 mg mL^−1^ GO dispersion, previously ultrasonicated for half an hour, onto the surface of the H-terminated Si(111) surfaces, and drying in air under heat (T = 50 °C) for 15 min. Two GO-coated silicon samples were prepared to allow for a spectroscopical characterization prior to and after electrochemical reduction, denoted GO_aq_ and GO_non-aq_ according to the electrolyte type used in their reduction (see below). According to the GO dispersion concentration and the aliquot volume used (the area of the drop being 0.3 cm^2^, and assuming the density of GO was close to 1.8 g cm^−3^ [51]), the thickness of the pristine GO layer on the silicon was estimated to be around 56 nm, corresponding to approximately 40 layers of individual GO sheets (considering a single GO sheet thickness of around 1.4 nm [52,53,54]). In the Raman spectroscopy characterization section of this work (vide infra), an alternative evaluation of the number of layers is given. The resulting GO_aq_ and GO_non-aq_ samples were used as working electrodes in a PTFE electrochemical cell, where the electroactive area was roughly the same as the drop-casted GO deposit (0.3 cm^2^).

The electrochemical studies in aqueous medium were performed in 1 M phosphate (K_2_HPO_4_/KH_2_PO_4_)-buffered saline (PBS) solution as the supporting electrolyte, with pH = 7.2 and a Ag/AgCl (sat.) reference electrode (E = 0.198 V vs. NHE). For the electrochemical studies in a non-aqueous medium a 0.1 M tetrabutylammonium hexafluorophosphate (TBAPF_6_, Sigma-Aldrich, Darmstadt, Germany) in dry CH_3_CN (Sigma-Aldrich, Darmstadt, Germany) solution was used. In this case, the employed reference electrode was a silver wire immersed in 0.01 M AgNO_3_/0.1 M TBAP in CH_3_CN (+0.544 V vs. NHE; +0.349 V vs. Ag/AgCl), separated from the main solution by a porous fritted glass, plus an agar plug. The resulting electrochemically reduced GO samples were denoted erGO_aq_ and erGO_non-aq_, respectively. In both types of electrolytes, a platinum wire counter electrode was used. It must be pointed out that for the sake of comparison, all the potentials in this study are referred to with respect to the Ag/AgCl (sat.) reference electrode. After electrochemical reduction, samples were extracted from the cell and thoroughly rinsed with distilled water. Nevertheless, in the erGO_aq_ sample traces of K from the PBS buffer were detected in the K 2p XPS region (around 294 eV BE).

All electrochemical measurements were performed using a BioLogic SP-150 potentiostat/galvanostat driven by the BioLogic EC-Lab^®^ software v11.27 (BioLogic, Seyssinet-Pariset, France) and a Teq4 potentiostat/galvanostat (NanoTeq, Buenos Aires, Argentina).

Raman spectra were measured at room temperature in backscattering geometry with an inVia Renishaw micro-Raman spectrometer (Renishaw, New Mills, Wotton-under-Edge, UK) equipped with an air-cooled CCD detector and super-Notch filters. An Ar^+^ ion laser (*λ_laser_* = 488.0 nm) was used, coupled with a Leica DLML microscope with a 5× objective. The resolution was 2 cm^−1^ and spectra were calibrated using the 520.5 cm^−1^ line of a silicon wafer. Raman spectra were acquired from several (6–10) different spots on the surface of the samples. *L_a_* (the average crystallite size) of the *sp*^2^ lattice was calculated using the following equation [55]:

(1) $$L_{a}\left(nm\right)\;=\;\left(2.4\times 10^{-10}\right)\lambda_{laser}^{4}{\left(\frac{I_{D}}{I_{G}}\right)}^{-1}$$

where *I_D_* and *I_G_* are the intensities of the Raman D and G bands, respectively, and *λ_laser_* is the laser wavelength line. All peaks were fitted with software routines that allowed symmetric and asymmetric Gaussian, Lorentzian or pseudo-Voigt profiles to simulate the spectra and extract single components.

XPS measurements were carried out using a modified Omicron NanoTechnology MXPS system (ScientaOmicron, Uppsala, Sweden) equipped with a monochromatic Al Kα (hν = 1486.7 eV) X-ray source (Omicron XM-1000) operating the anode at 14 kV and 16 mA. The C 1s photoionization region was acquired using an analyzer pass energy of 20 eV and take-off angle of 21° with respect to the sample surface normal. The experimental spectra were theoretically reconstructed by fitting the secondary electrons background to a Shirley function and the elastic peaks to pseudo-Voigt functions described by a common set of parameters: position; full-width at half-maximum (FWHM); and Gaussian–Lorentzian ratio. The relative amounts of the different oxygenated functional groups were determined through area ratios with an uncertainty of ±10%.

## 3. Results and Discussion 

**Scheme 1:**
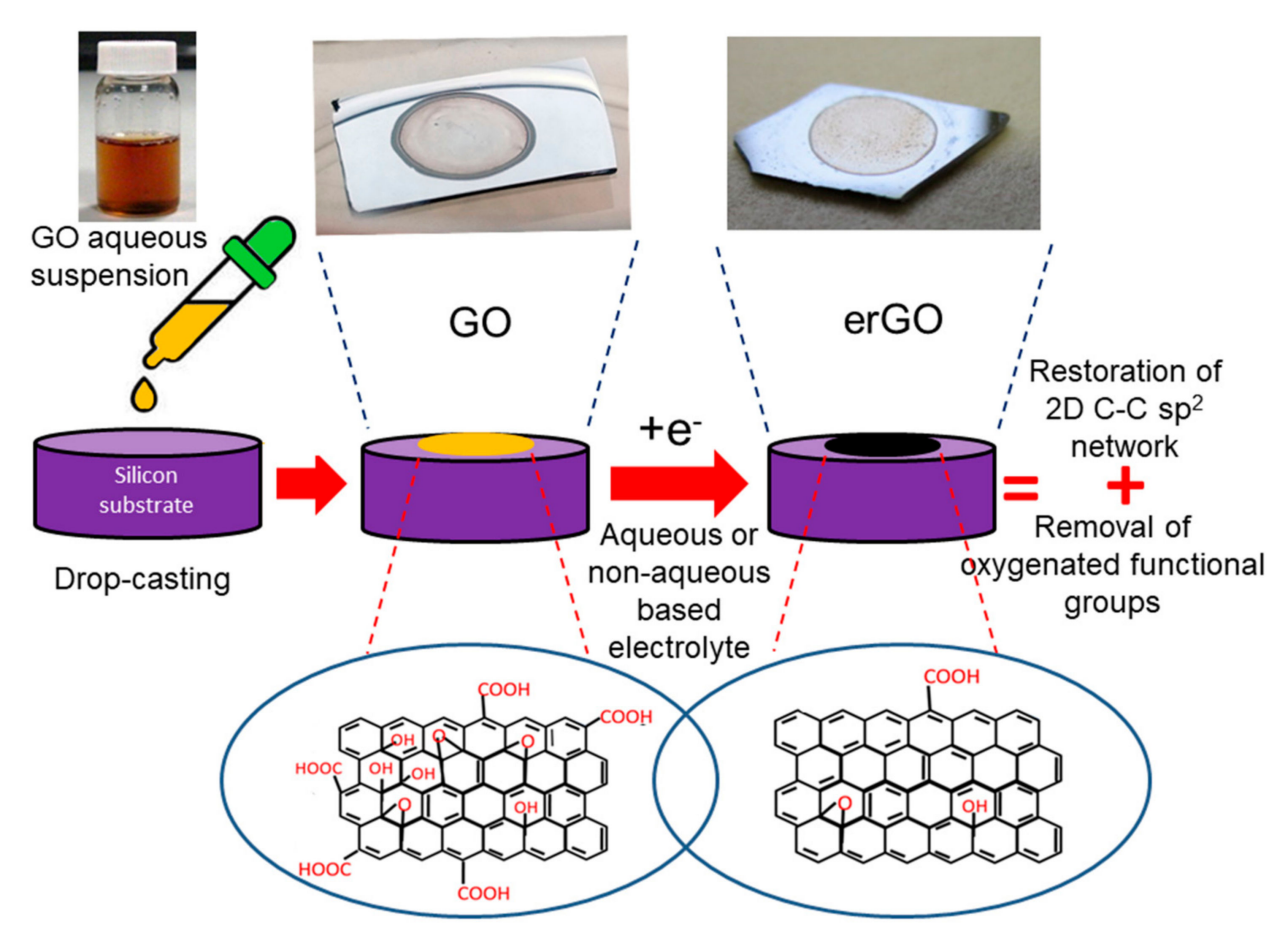
Schematic illustration of the preparation route followed to obtain the erGO layers on a H-terminated n-type Si(111) substrate in both the aqueous and non-aqueous electrolytic media.

The electrochemical reduction of the GO layers was carried out through a two-step route, involving: (i) drop-casting the GO sheets onto a silicon electrode surface; then (ii) electrochemical reduction using a standard three-electrode electrochemical system in the presence of an aqueous or non-aqueous electrolyte. Scheme 1 depicts this procedure in the production of electrochemically reduced GO (erGO) layers on a silicon surface.

**Figure 1:**
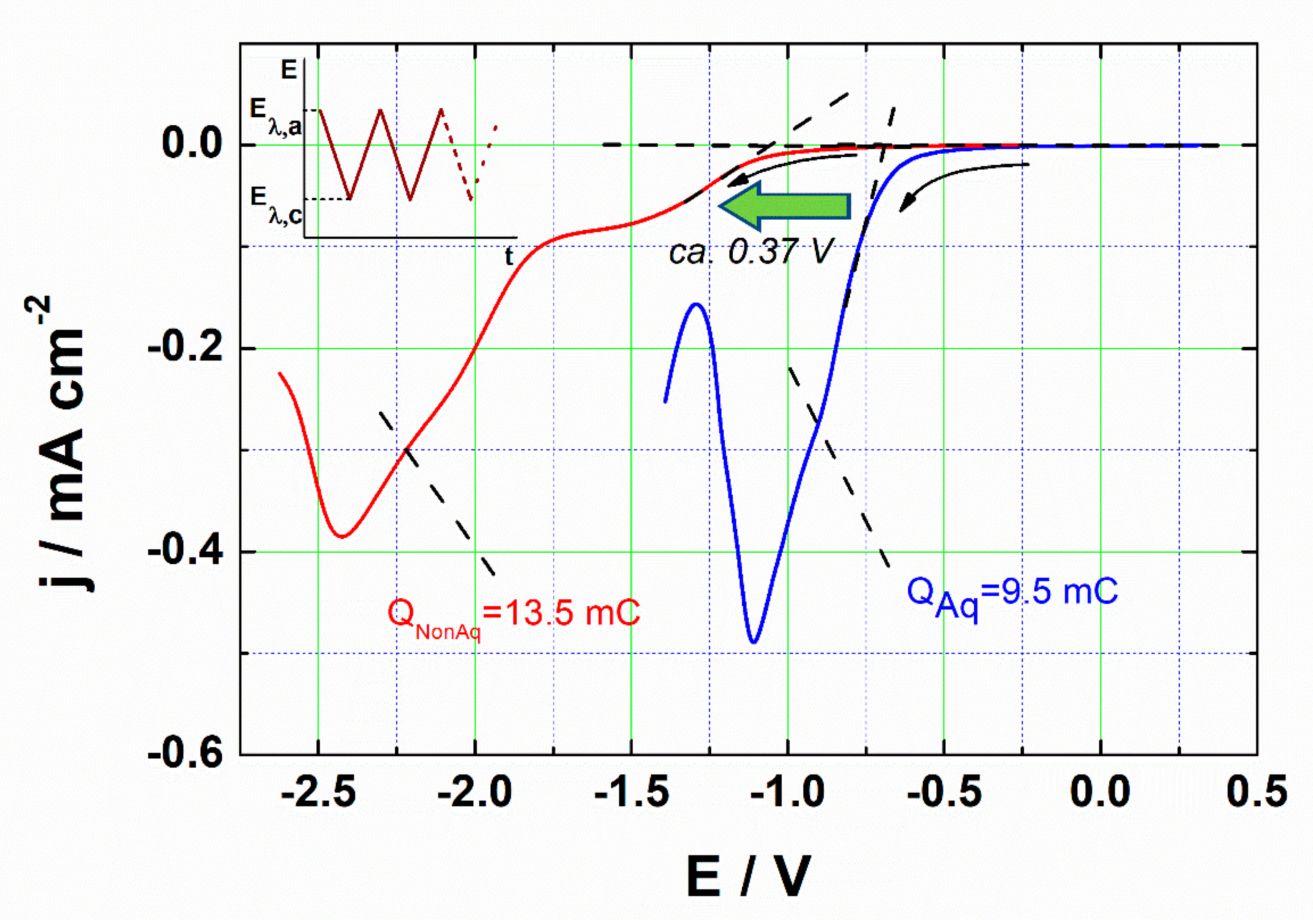
Voltammetric response recorded during the electrochemical reduction of a GO sample in both an aqueous (1.0 M pH = 7.2 PBS buffer solution) and a non-aqueous (0.1 M TBAPF_6_/CH_3_CN solution) electrolytic medium (blue and red solid lines, respectively). The potential scan rate was 20 mV s^−1^. The black solid arrows indicate the potential scan direction. The amount of electric charge (Q) obtained by the integration of the corresponding voltammetric curves is also indicated. Inset: potential versus time program for the potential cycling (PC) electrochemical procedure showing the forward and reversed linear potential ramp within a potential range.

In order to attain the reduction of the supported GO layers, a potential cycling (PC) electrochemical technique was used. To this end, the applied potential (E) of the working electrode was linearly varied with time at a given scan rate (*ν*) from an initial anodic potential (*E_λ,a_*) to a reversal cathodic one (*E_λ,c_*), and the sweeping cycles were repeated for a certain number of times (see inset of Figure 1). In this work, the electrochemical study was carried out for different potential scan rate values, whereas the XPS analysis has been applied to samples prepared at 20 mV s^−1^.

Figure 1 shows the voltammetric response recorded during the first potential scan (toward cathodic potentials, i.e., the forward scan) of the PC electrochemical reduction of a GO-coated silicon electrode in both aqueous (*E_λ,a_* = 0.30 V; *E_λ,c_* = −1.4 V) and non-aqueous media (*E_λ,a_* = 0.25 V, *E_λ,c_* = −2.6 V) at a scan rate of 20 mV s^−1^.

**Figure 2:**
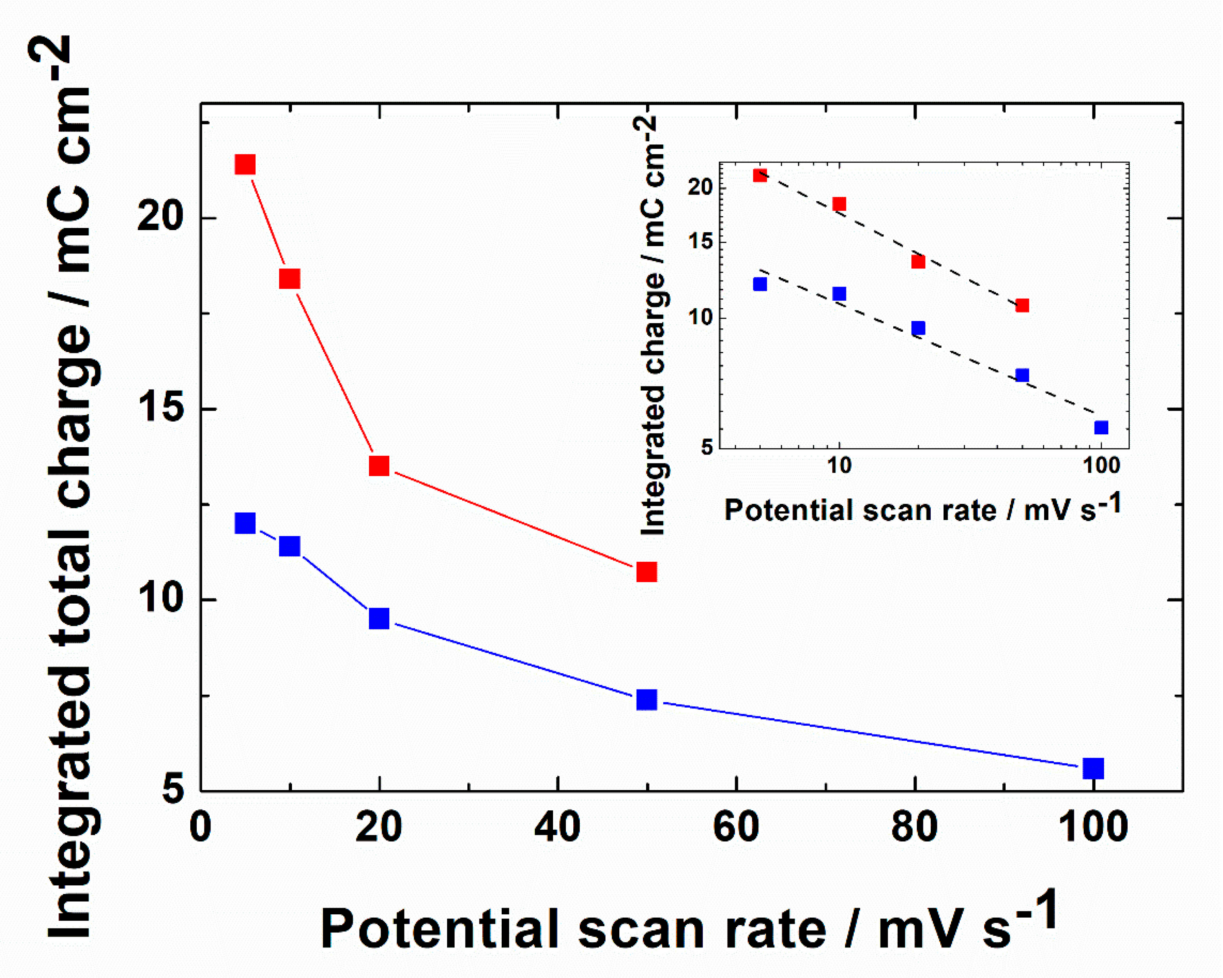
Integrated charge of the cathodic voltammetric wave recorded during the electrochemical reduction of GO samples in both aqueous (1.0 M pH = 7.2 PBS buffer solution) and non-aqueous (0.1 M TBAPF_6_/CH_3_CN solution) electrolytic media as a function of the potential scan rate (blue and red filled squares, respectively). Inset: same plots in a double logarithm format. The black dashed lines correspond to the linear fitting.

As it was previously reported that in further consecutive cycles the electrochemical features of interest are absent in the same potential scan range [40,48], only the first cathodic potential scan is presented in the following. The voltammetric profiles obtained in both aqueous and non-aqueous media exhibit a well-defined irreversible broad reduction peak attributed to the reduction of the surface-bound OFGs on GO [40,48]. These broad peaks represent the contribution of several irreversible electrochemical processes. These facts are typical of GO materials containing different OFGs, primarily the epoxyl and carbonyl groups [40,56,57,58,59,60,61], and can generally be related to the existence of several consecutive electrochemical processes [50,61]. In Figure 1 it can be seen that the cathodic peak potential of the electrochemical feature observed in the non-aqueous medium (−2.43 V) was much more negative than the cathodic peak potential recorded in the aqueous medium (−1.11 V). From this it can be inferred that the oxygen functionalities attached to GO were thermodynamically more stable in the non-aqueous medium than in water [48]. Moreover, it can be appreciated that a non-aqueous medium has the advantage of exhibiting a broader potential window compared to an aqueous medium, thus allowing it to reach more negative reduction potentials (i.e., *E_λ,c_* = −3.0 V compared to *E_λ,c_* = −1.3 V, see Figure 1). As has been said above, it is commonly accepted that on GO nanosheets the carboxyl (–COOH) and carbonyl (C=O) groups are mainly located on *sp*^2^ hybridized carbon atoms placed at the edges of GO nanosheets, while the phenolic hydroxyl (–OH) and epoxy (C–O–C) groups are primarily located on *sp*^3^ hybridized carbon atoms placed on the basal plane [14,62]. Moreover, in relation to their chemical reactivity, the OFGs located on the basal planes and on the edges and basal plane defects are characterized as weak and strong OFGs, respectively [14,62,63]. For instance, the functional groups on the edges, such as carboxylic acids, are much more stable and less likely to react first. Therefore, the highly negative potential was expected to help overcome the energy barriers to their reduction, as is further presented and discussed below. Figure 1 shows that the onset potential of the voltammetric reduction broad peak in the aqueous case was placed at *E_on, aq_* = −0.69 V, whereas in the non-aqueous medium the onset potential moved toward more cathodic potentials of about 0.37 V, i.e., *E_on, non-aq_* = −1.06 V, indicating a less favorable reduction process in this organic medium. The amount of electric charge (Q) obtained by the integration of the corresponding voltammetric curves could be related to the removed amount of the different OFGs by the Faraday’s law of electrolysis. As is indicated in Figure 1, the reduction charge corresponding to the electrochemical non-aqueous process was greater than that of the aqueous one—i.e., Q_non-aq_ = 13.5 mC vs. Q_aq_ = 9.5 mC—indicating a larger degree of OFGs reduction in the first case. The wide and complex wave-shape of the voltammetric response in both studied media hampered the analysis of the (peak) current as a function of the time-scale (potential scan rate). To avoid this issue, in a first approach, the integral response (charge) as a function of the potential scan rate was considered. Figure 2 shows the relationship between the integrated amount of charge corresponding to the graphene oxide reduction voltammetric wave and potential scan rate for studies carried out in both aqueous and non-aqueous media.

It can be appreciated that in the case of the erGO_non-aq_ sample (red dots), the involved electric charge was larger than that employed in the production of the erGO_aq_ sample, irrespective of the potential scan rate. This clearly suggests that a larger amount of OFGs was reduced when the reduction was carried out in a non-aqueous medium. As shown in Figure 2, the integrated charge was strongly affected by the potential scan rate. The net charge was proportional to the inverse of the potential scan rate, indicating a sluggish and kinetic-controlled process on the graphene oxide reduction [64,65]. Furthermore, in order to explore the possibility of simple power law dependence, the double logarithmic format was chosen and depicted as inset in Figure 2. It can be appreciated that *log Q* scales linearly with *log ν*. The slopes of these plots are −0.18 (r = 0.993) and −0.21 (r = 0.999) for the aqueous and non-aqueous cases, respectively. These values are not as large as the value of −0.5 characteristic of a diffusionally controlled process, whereas they are closer to the value of zero, characteristic of an ideal surface-confined redox couple, as expected for OFGs decorating the 2D graphene oxide network [66,67]. It should be noted that the reaction path of the electrochemical reduction to obtain erGO is not exactly known and few studies have focused so far on the elucidation of the electrochemical reduction mechanism [10,41,63]. Reasonably, the electrochemical reduction of GO is expected to take place in several steps, due to the varied chemical structure of graphene oxide nanosheets [15,40,48]. In fact, as reported in Figure 1, in the aqueous and non-aqueous media, the *j*–*E* voltammetric response exhibited a broad band feature, as an indication of the numerous types of oxygen functionalities present, which were reduced at different potentials (merged into one single broad band response).

**Figure 3:**
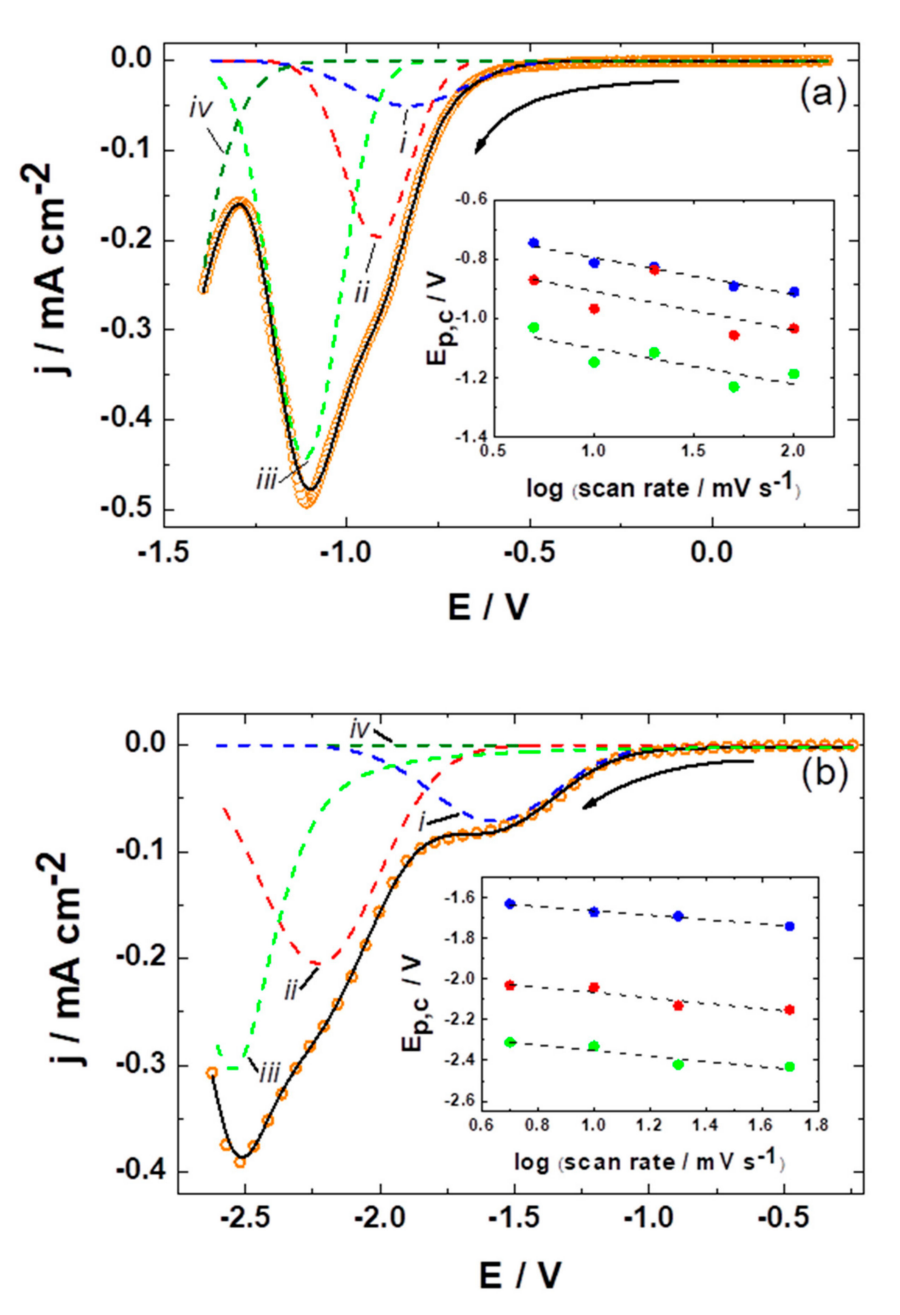
Deconvolution of typical cathodic waves observed during the electrochemical reduction of a GO sample in: (**a**) 1.0 M pH = 7.2 PBS buffer solution and (**b**) 0.1 M TBAPF_6_/CH_3_CN solution at a scan rate of 20 mV s^−1^. The graphs show: experimental current-potential data (orange open circles); deconvolution peaks (coloured dashed lines, from *i* to *iii* as indicated); and a simulated envelope voltammogram (black solid line). The solvent contribution curve *iv* is also depicted. Insets: cathodic peak potential versus logarithm of the potential scan rate for the different peaks involved in the deconvolution process (peaks *i*, *ii* and *iii* correspond to blue, red and green filled circles, respectively). The black dashed lines correspond to the linear fitting.

To gain insights into the electrochemical processes occurring during the electrochemical reduction of the GO layers, the different electrochemical contributions to the cathodic broad peak were separated by means of a deconvolution analysis applied to the voltammetric curves obtained at a potential scan rate of 20 mV s^−1^ (see Figure 3; for other voltammetric studies recorded at different potential scan rate values in an aqueous medium, please see our previously published work [41], and for studies carried out in a non-aqueous medium, see Figures S1–S3 in the Supplementary Material of the present work).

The voltammograms were treated by numerical deconvolution with a non-linear fitting procedure of the experimental data using Gaussian model functions [68,69,70]. In both aqueous and non-aqueous cases, the observed broad peak was deconvoluted into three individual main peaks: *i*, *ii* and *iii* (which can be assigned to the electrochemical reduction of the different OFGs present on the GO nanosheets) and a fourth one, *iv*, which appears at more cathodic potentials, which can be associated with the electrolyte electrochemical decomposition. Therefore, in the following description only the three major components (*i* to *iii*) are addressed. It can be appreciated that in both aqueous and non-aqueous media, three of the four resulting peaks (*i* to *iii*) exhibited a very important contribution to the voltammetric wave. Such features probably represent a very specific stage of the electrochemical reduction process, which is not a straightforward matter to assign [10,18,19,41,63]. Based on the relative stability of each of the OFGs, one can expect that a possible reduction sequence would be as follows: epoxy, carbonyl, hydroxyl then carboxyl. Since hydroxyl and carboxyl groups are expected to require significantly more energy for their reduction [60,71], we attributed peaks *i*, *ii* and *iii* to reduction events related to GO domains rich in epoxy and carbonyl groups. More specifically, as will be reported in a forthcoming paper, epoxy groups in the basal plane of GO may display significantly different redox potentials depending on their chemical environment, which could explain the multiplicity of the redox features composing the reduction band resulting from CV.

Since the potential values (*E_p,c_*) of the deconvoluted individual peaks appear to shift to more cathodic values as the potential scan rate increases (please see our previously published work [41] and the SM of the present work for studies carried out in aqueous and non-aqueous media, respectively), a further evaluation of the influence of the scan rate on the cathodic peak potential was carried out. The variation of *E_p,c_* with the logarithm of the scan rate, *log ν*, for the different deconvoluted peaks is shown in the insets of Figure 3 for both aqueous and non-aqueous medium cases. It can be appreciated that the *E_p,c_* scaled linearly with *log ν*, which allowed us to determine the value of the overall electron transfer coefficient α*n* (where α is the transfer coefficient and *n* is the number of electrons transferred for the oxidation/reduction process) through Laviron’s approach. According to Laviron’s description of an irreversible electrochemical process [65,72], *E_p,c_* is related to the potential scan rate *ν* by:

(2) $$E_{p,c}\propto -\frac{2.303RT}{\alpha nF}logv$$

where *n* is the number of electrons transferred, *α* is the transfer coefficient and the other symbols have their usual meanings. The transfer coefficient physically provides an insight into the way the transition state is influenced by the voltage, with typical values in most electrochemical systems lying between 0.3 and 0.7 [65,72]. Therefore, on the basis of Equation (2), the values of *αn* could be calculated from the slopes of the *E_p,c_* vs. *log ν* plots. Both slope and *αn* values are reported in Table 1, with the former ranging from 106 to 134 mV/dec (with a mean value of ca. 120 mV/dec). These ranges of values point to an electrochemical reduction process in which the first charge transfer is the determining rate step (without involving diffusional processes) [65,72]. This is typical of ideal electrode surface-confined redox active groups, such as those related to OFG moieties decorating the GO nanosheets. In these cases, an apparent standard electron transfer rate constant, *k_app_*, can be determined using Laviron’s formulation [72,73,74]:

(3) $$k_{app}=\frac{\alpha nFv}{RT}$$

where *αn* is the overall transfer coefficient [75], *ν* is the potential scan rate and the other symbols have their usual meanings. From this treatment, and introducing the obtained *αn* values portrayed in Table 1 and a potential scan rate of 50 mVs^−1^ (the common highest value assayed in both media, see Figure 2), different apparent rate constant values were found, collected in Table 1. 

### 3.1. XPS Characterization

**Figure 4:**
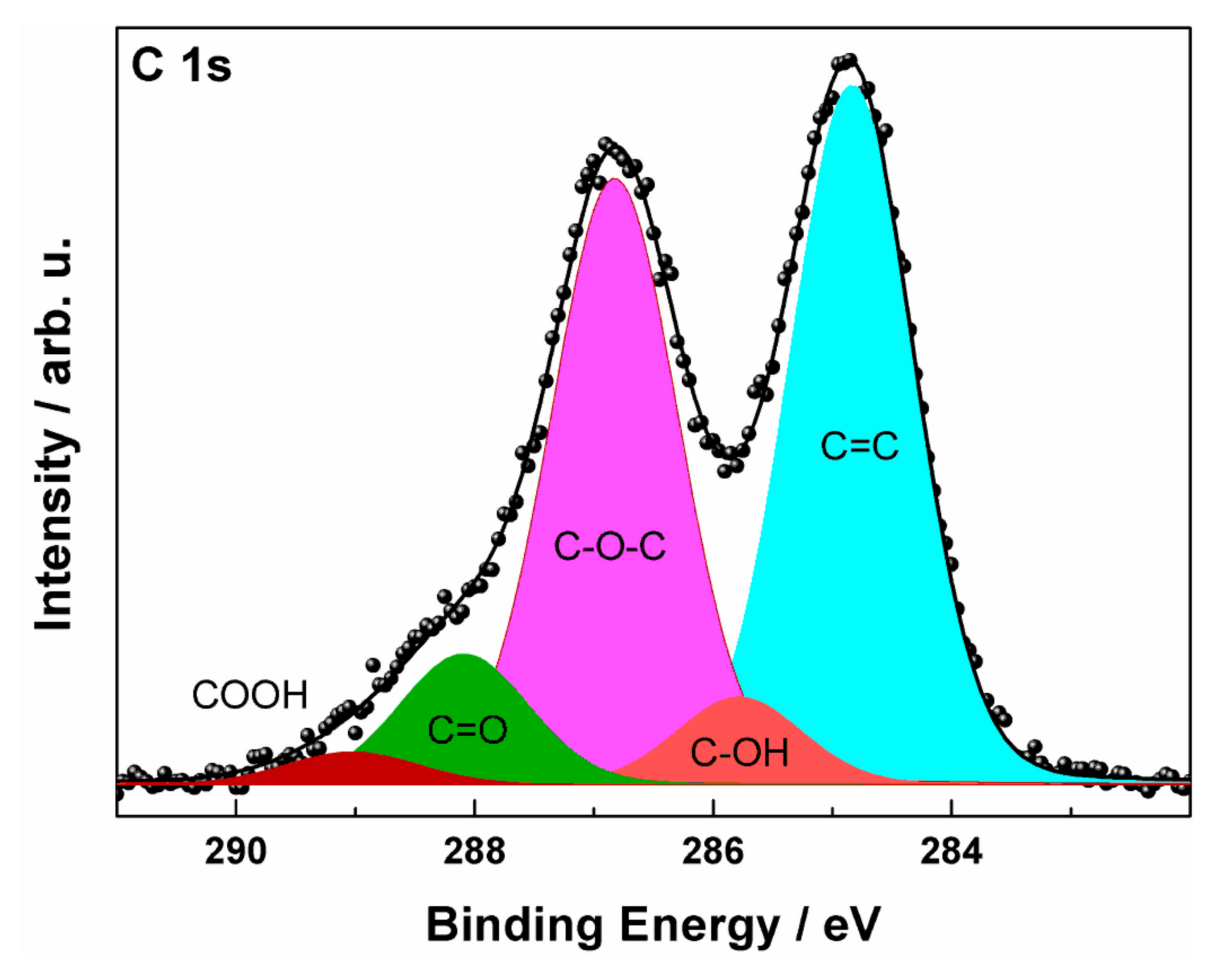
C 1s XP spectrum of a pristine GO_aq_ sample. Raw data are displayed with dots, while a fitting reconstruction is represented by a continuous line and color-filled curves corresponding to the various OFGs.

X-ray photoelectron spectroscopy was applied to two distinct GO samples to investigate the chemical composition variations prior to (GO_aq_ and GO_non-aq_) and after electrochemical reduction in aqueous (erGO_aq_) and non-aqueous (erGO_non-aq_) media. The C 1s ionization region was selected as particularly diagnostic of the changes in quantity and nature of OFGs. Figure 4 shows the C 1s spectrum of GO_aq_ as representative of the GO deposit prior to electrochemical reduction (see Figure S4 in the Supplementary Material for the C 1s spectrum of GO_non-aq_).

**Figure 5:**
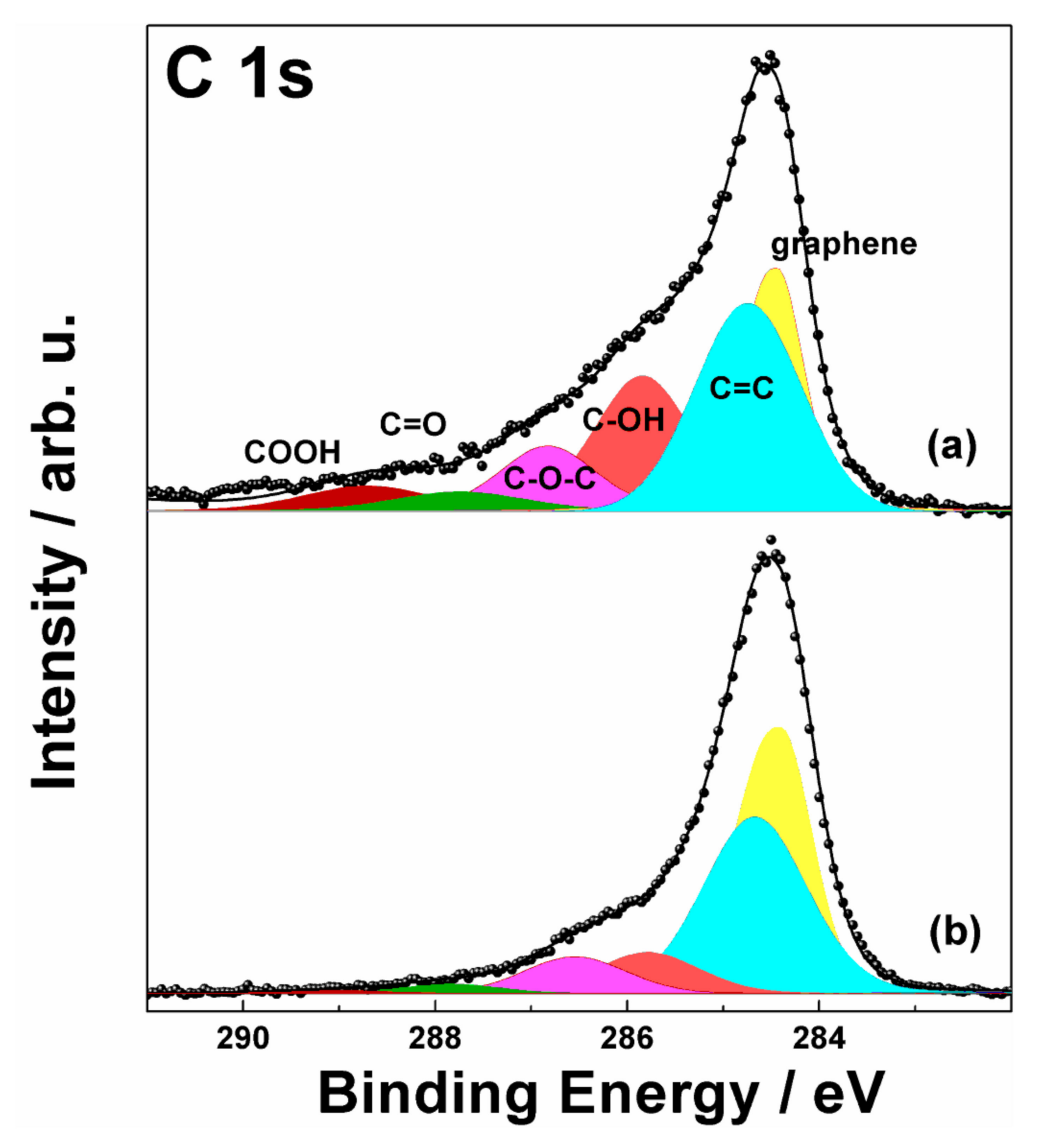
C 1s XP spectra of (**a**) erGO_aq_ and (**b**) erGO_non-aq_ samples obtained at 20 mV s^−1^ potential scan rates. Raw data are displayed with dots, while a fitting reconstruction is represented by a continuous line and color-filled curves corresponding to the various OFGs.

The typical spectral contributions of GO arising in this region are those related to the carbon atoms surrounded by different chemical environments [40,76,77], attributed C=C groups with nearly localized characteristics, C–OH (hydroxyl), C–O–C (epoxyl), C=O (carbonyl, quinonyl) and COOH (carboxyl) OFGs [76,77,78] (see Table 2). According to Figure 4 and Table 2, it is apparent that the dominant OFG in pristine GO was the epoxy group on the basal plane, as expected according to Hummers’ method used by the supplier for its synthesis. Upon electrochemical reduction (Figure 5, Figure S5 and Figure S6) the same C 1s components could be found, but a further peak was needed to allow for a satisfactory convergence of the fitting procedure. This additional feature (i) was narrower than the others, (ii) displayed an asymmetry on the high-BE side, and (iii) was shifted to a low BE with respect to the C=C component in pristine GO. According to these characteristics, this contribution can be assigned to C=C groups within an extended network of conjugated π bonds, such as that resulting from the restoration of the graphene structure after the elimination of oxygenated moieties [40,48,78].

According to the area ratios (see Table 2) between the different peaks in the C 1s spectra of the erGO_aq_ and erGO_non-aq_ samples, one can see that upon the reduction of GO the major abatement of OFGs concerned the epoxy moieties, followed by the C=O groups.

**Figure 6:**
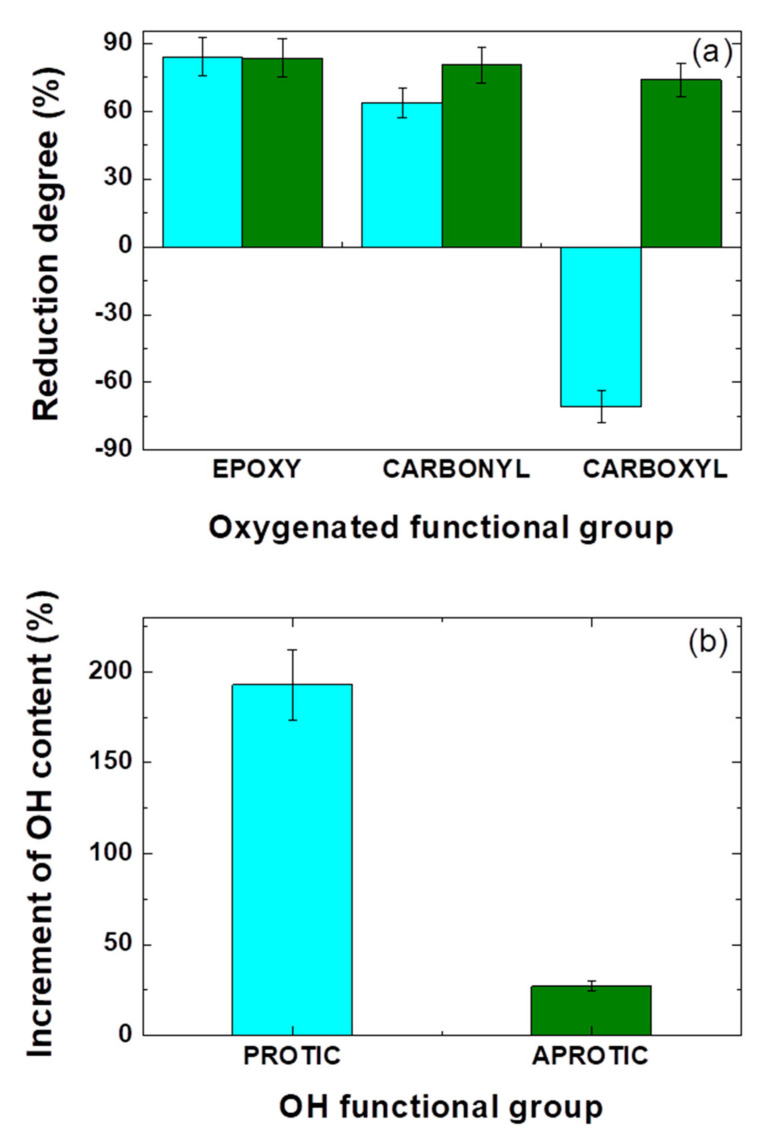
(**a**) Relative removal degree percentage of three different OFGs (as indicated) and (**b**) increment degree percentage of the hydroxyl OFG after the electrochemical reduction process of typical GO-coated Si electrodes in 1.0 M pH = 7.2 PBS buffer solution and 0.1 M TBAPF_6_/CH_3_CN solution (light cyan and olive columns, respectively). The potential scan rate was 20 mV s^−1^ over an entire electrochemical process of five cycles. In all cases, these OFG content values were calculated from the C 1s XPS spectra.

An estimate of the amount of the removal (reduction degree in percent) of the different OFGs initially present in the GO_aq_ and GO_non-aq_ nanosheeets, *R_OFG_*(%), was calculated through the following equation:

(4) $$R_{OFG}=\frac{X_{OFG}^{GO}-X_{OFG}^{erGO}}{X_{OFG}^{GO}}\times 100$$

where $X_{OFG}^{GO}$ and $X_{OFG}^{erGO}$ are the percentage amounts of the corresponding OFG on the GO surface before and after the electrochemical reduction, respectively. On the basis of the $X_{OFG}^{GO}$ and $X_{OFG}^{erGO}$ values extracted from the XPS results presented in Table 2, comparison plots of the reduction degrees for the main OFGs, i.e., epoxy, carbonyl, carboxyl and hydroxyl are presented in Figure 6 and Table S1. These results show two different and opposite behaviors corresponding to the various OFGs. In fact, as anticipated above, the epoxy and carbonyl OFGs were both abated, whereas the hydroxyl content increased in both the aqueous and non-aqueous media (Figure 6). On the other hand, the carboxyl OFG content increased in the aqueous medium and decreased in the non-aqueous one (Figure 6). More specifically, the reduction degree of the epoxy OFG was similar in both electrolytic media, whereas use of the non-aqueous medium was more efficient for the reduction of the carbonyl OFG.

As to the hydroxyl group, the bar graph in Figure 6 shows that electrochemical reduction led to an increase in the intensity of the corresponding XPS C 1s component in both types of electrolytes used. This increase was found to be far more pronounced for the sample reduced in the protic environment, in agreement with other previously published papers [79,80]. This increment was already also documented by us [41] and is probably related to relatively stable hydroxylated intermediate steps [81] in the electrochemical reduction reaction path involving a protic medium. In the aprotic medium, the reduction mechanism has not been clarified yet, and formally the absence of Brønsted-type acidic species is expected to induce a different reaction path. On the other hand, we detected a small yet observable increase in the OH content also in the erGO_non-aq_ sample, which we speculate could be due to the weak H^+^-donor characteristic of the solvent used, acetonitrile (pKa = 31.1 in dimethylsulfoxide [82]). Some authors have also argued that, since electroreduction produces a large amount of C–H defects, these are available to re-oxidation in aqueous solutions, likely producing C–OH during the anodic run of the CV [18,80].

In the particular case of the COOH group, an increase in its content has previously seldom been reported using electrochemical reduction of GO in a protic medium [10,71,80] and very mild thermal annealing of GO nanosheet powders [83]. The formation of COOH groups was proposed by some authors as a possible intermediate step in the chemical reduction of GO exerted by strong bases, such as NaOH, eventually leading to complete decarboxylation with CO_2_ evolution [84]. Furthermore, other authors reported a COOH increment as a result of a photoreduction triggered by hydroxyl ions within the vacancy defects of the GO basal plane. In our case, we hypothesize that this increment may have stemmed from the action of freshly electrochemically generated OH^−^ ions at the surface of GO when the cathodic potential reached the limit of water decomposition (~−1.5 V vs. Ag/AgCl), thus leading to the localization of carboxyls at the edges of nano-holes, resulting from the electrochemical reduction of GO [85]. 

Although no direct proof is available to support this hypothesis, the fact that this “carboxylation” was limited to the erGO_aq_ sample constitutes a possible step in that direction. 

As to the carboxyl abatement in an organic medium, this is an unprecedented result considering the use of an electrochemical reduction route. Though no explicit electrochemical features corresponding to COOH reduction could be identified in the voltammogram related to the erGO_non-aq_ sample, it is likely that this process may have partially been shaded by the solvent decomposition occurring below −2.7 V. The abatement of carboxyl groups turns out to be particularly relevant due to their reported negative role in the voltammetric response of the resulting erGO materials [19].

**Figure 7:**
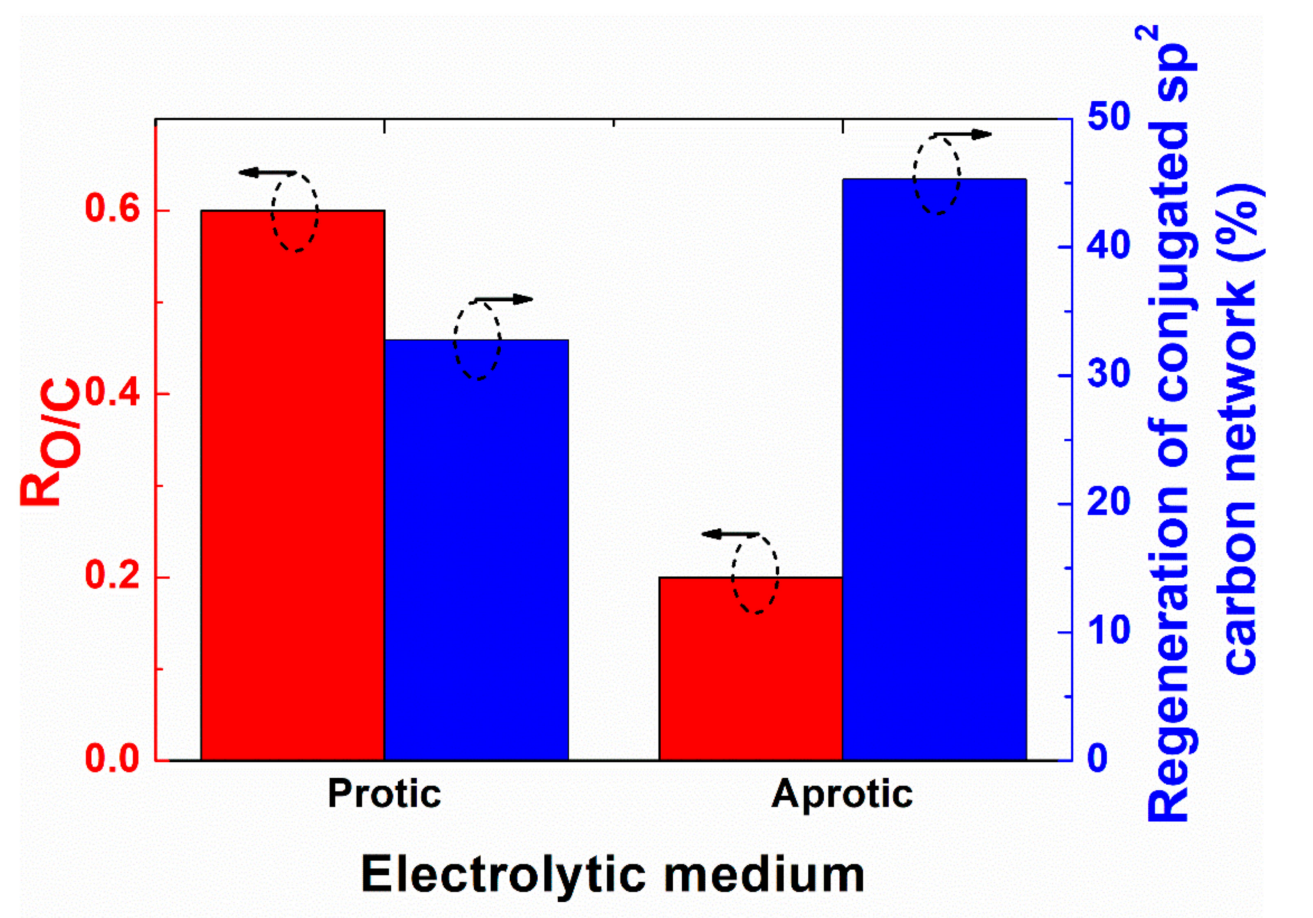
Oxygen-to-carbon ratio (R_O/C_) and regeneration of conjugated *sp*^2^ carbon network percentage after the electrochemical reduction process of typical GO-coated Si electrodes in 1.0 M pH = 7.2 PBS buffer solution (protic) and 0.1 M TBAPF_6_/CH_3_CN solution (aprotic). The potential scan rate was 20 mV s^−1^ over an entire electrochemical process of five cycles. In all cases these data were calculated from the C 1s XPS spectra.

The oxygen-to-carbon ratio (R_O/C_) was determined as an additional independent test of the GO reduction extent, considering that a decrease in R_O/C_ corresponds to a more efficient reduction. In this work, the R_O/C_ were calculated using the C 1s curve-fitting results according to the procedure previously described in [86,87] (see the SM for details). The obtained R_O/C_ values for the erGO_aq_ and erGO_non-aq_ samples are depicted in Figure 7. It can be appreciated that more oxygen-containing functional groups were removed during the reduction in the non-aqueous medium (R_O/C_ = 0.20) compared to the aqueous one (R_O/C_ = 0.60). The R_O/C_ value obtained for the erGO_non-aq_ sample is lower than the ones previously reported for electrochemically reduced GO in an acidic medium (R_O/C_ = 0.26) and in a proton-free organic electrolyte (R_O/C_ = 0.56) [18], and approximately the same as that obtained from combined electrochemical and photochemical routes (R_O/C_ = 0.21) [19] and from chemical, biological and thermal-based reduction approaches [5]. 

Figure 7 also shows that the graphitic carbon *sp*^2^ domains were restored to a larger extent in the non-aqueous electrolyte when compared to that in the aqueous one. The efficient reduction of pristine GO nano-sheets in the aprotic electrolyte is therefore evidenced by the coupling of a good R_O/C_ value with a good restoration of *sp*^2^ network.

### 3.2. Raman Spectroscopy Characterization

GO and erGO samples were further characterized by Raman spectroscopy, with the aim of evidencing structural order changes upon electrochemical reduction treatments [54,58,88,89].

**Figure 8:**
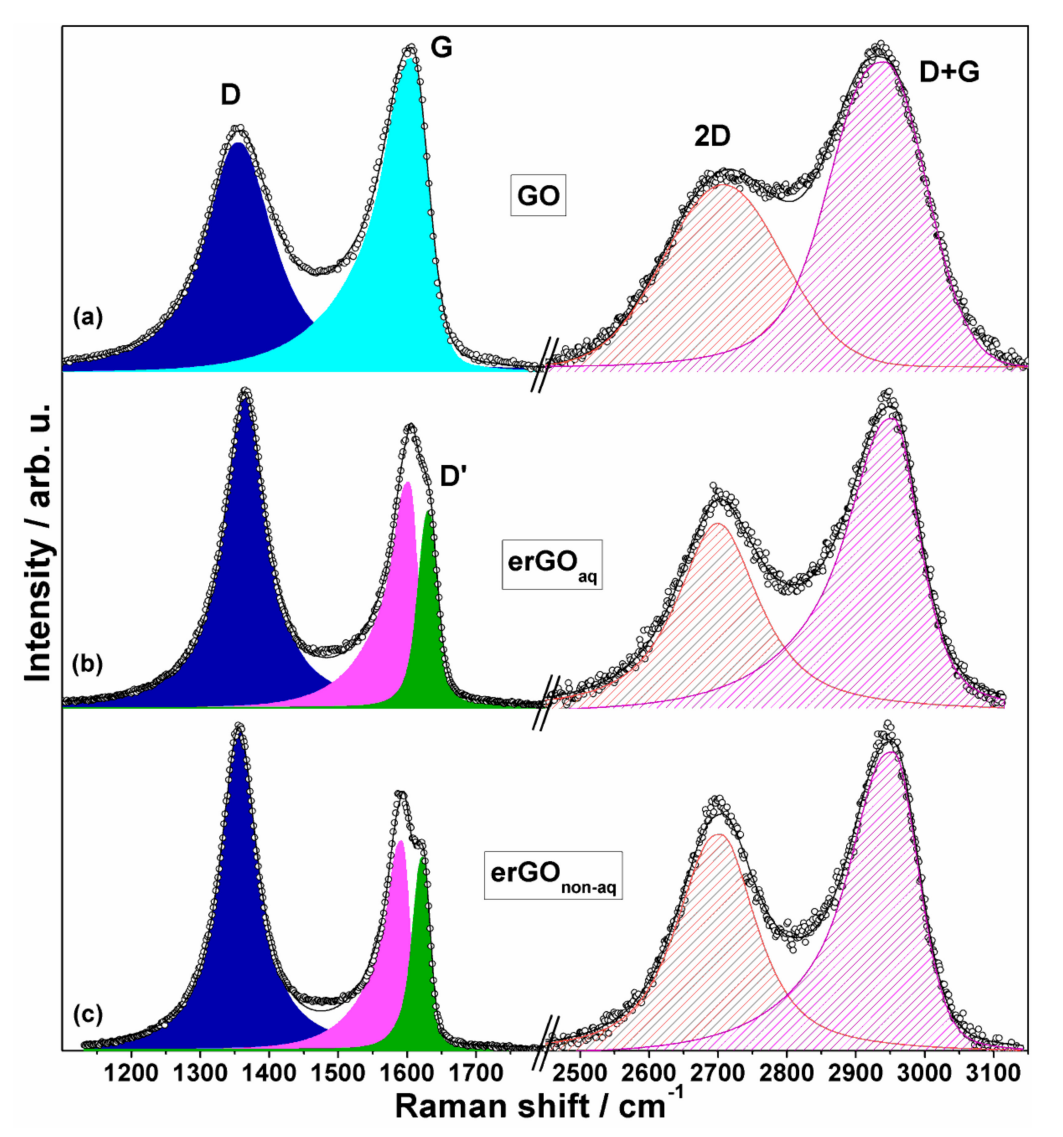
Experimental (dots) and theoretically reconstructed (colored and shaded curves) Raman spectra of (**a**) GO, (**b**) erGO_aq_, and (**c**) erGO_non-aq_. Both low energy (left panel) and high energy (right panel) features are displayed. The potential scan rate for the electrochemical reduction of GO samples was 20 mVs^−1^.

Figure 8 shows the most significant features in the Raman spectra of the GO (GO_aq_ is reported as representative of both pristine GO samples) and erGO samples—i.e., the D and G bands (within the range 1200–1700 cm^−1^) and the weaker 2D and D + G bands (within the range 2600–3000 cm^−1^), which are typical of graphene-based carbon compounds [90,91]. All the bands were theoretically reconstructed with a curve fitting based on model functions, which required in the case of the erGO samples the presence of a second component attributed to the D’ mode, partially overlapping with the G peak. 

The G band arises from the E_2g_ in-plane vibration of the *sp*^2^ carbon network and is usually taken as diagnostic of the degree of graphitization. The D band can be attributed to local defects, vacancies and grain boundaries, since it stems from the A_1g_ κ-point phonons breathing mode [89,91,92] and, together with the 2D band, is also related to vibrations of *sp*^3^ carbon atoms [90,91]. 

In the spectra reported in Figure 8, a variation of the relative intensities of the two low-energy bands is apparent upon going from pristine GO to the erGO samples. In the literature, the intensity ratio of the D and G peaks (*I_D_/I_G_*) has been widely reported either in the form of the peak height or area ratio as an indication of the disorder degree of graphitic materials [55,92,93,94,95]. 

In all the investigated samples, we adopted a curve-fitting reconstruction approach to the Raman spectra in order to extract more precise band parameters, such as the position and full-width-at-half-maximum (FWHM) [96]. 

The peak-fitting of the band located around the 1600 cm^−1^ peak required one single, asymmetric Lorentzian curve for GO (G band), while two Lorentzian curves (G and D’ bands, the former symmetric and the latter asymmetric) were needed for erGO (Figure 8b, green and magenta curves). This difference, due to the presence of the D’ component in the erGO samples, hampered a straight comparison between the GO and erGO samples; therefore, a conventional peak height ratio analysis of the *I_D_/I_G_* ratio is followed henceforth.

As reported in Table 3, the *I_D_/I_G_* ratio tended to increase in the order GO < erGO_aq_ < erGO_non-aq_, pointing to the formation of new *sp*^2^ C-conjugated domains in the context of a general increase in the defectivity upon the removal of oxygen-containing groups [97], this effect being more pronounced when the non-aqueous medium was used for the electrochemical reduction. To this end, the relation proposed by Pimenta et al. [55] was used to evaluate the lateral average dimension of graphitic domain sizes (L_a_), obtaining the trend GO > erGO_aq_ ≈ erGO_non-aq_. It turned out, then, that upon reduction an extension of the area covered by graphitic domains occurred, although the single domains in both erGO samples appeared to be reduced to the same dimensions and separated by an increased number of defects. 

In addition, it has to be noted that the D peak significantly narrowed upon electrochemical reduction according to the trend GO > erGO_aq_ > erGO_non-aq_, in accord with values reported for reduced GO layers [98]. According to the FWHM of the Raman D peak, the electrochemical reduction approach based on the non-aqueous electrolyte appears to attain the highest degree of reduction, with only a low fraction of carbon atoms in a non-*sp*^2^-hybridized state [99].

Furthermore, a red shift from 1607 to 1592 cm^−1^ of the G band upon reduction from GO through erGO_non-aq_ could be seen, with the erGO_aq_ sample being very close to GO. This is another diagnostic feature of the partial recovery of the hexagonal *sp*^2^ network in graphene based materials [100].

On the right side of Figure 8 the high-energy region between 2400 and 3150 cm^−1^ of the Raman spectra is shown for all the investigated samples. In this range the 2D peak appeared around 2700 cm^−1^ and was associated with second order zone-boundary phonons arising from double-resonance Raman scattering [101]. From the FWHM value of the 2D peak, the number of layers in few-layer graphene may be inferred [91,102,103]. Using the approach given by Peng et al. [52], based on the correspondence between 125 cm^−1^ and 7–8 layers, a slight decrease from 11 to 7 layers could be determined upon going from GO through erGO_non-aq_. Such a decrease in the thickness of the graphene deposit on the silicon electrode could be explained by a partial chemical exfoliation induced by the removal of oxygenated functionalities during reduction [104,105] that also results in a smoother morphology, as we already pointed out in the case of reduction in an aqueous medium [40].

Overall, the results from the Raman spectroscopy characterization point to the attainment of a more efficient reduction of GO when a non-aqueous electrolyte was used instead of an aqueous one. In fact, a higher number of new graphitic domains were formed in the erGO_non-aq_ samples compared to the erGO_aq_ samples, though they both showed similar lateral dimensions [93,94,101,106]. On the other hand, the removal of oxygenated functional groups, while being more efficient in erGO_non-aq_, also led to an increased structural disorder and density of defects [93].

## 4. Conclusions

The issue of optimizing the available recipes for an improvement of the scientifically and technologically relevant graphene–Si(111) interface has been addressed in this study by means of a combination of the most suitable techniques, notably XPS and Raman spectroscopy. Two electrochemical processes were followed, implying aqueous or organic media, leading to GO reduction. The adopted procedure was to assign each XPS C 1s component to specific functional groups, identify reduction features in cyclic voltammetry from peak deconvolution and monitor the fate of such groups in the two distinct conditions. This allowed for an in-depth critical comparison between the two interfaces. Conclusive evidence for the superior quality of a non-aqueous vs. aqueous GO to erGO reduction on Si(111) has been reached with the further help of Raman spectra, with the aim of establishing a better graphene final deposit. Further studies are in progress for exploiting additional effects of the GO parameters on the final interface, with a view to reaching the stage of a fine tuning of such an interface in a specifically required application. 

## Figures and Tables

**Table 1:** Slope values obtained from the *E_p,c_* vs. *log v* plots depicted as insets in Figure 3 and parameters obtained from Laviron’s approach applied to the electrochemical reduction of GO in both aqueous and non-aqueous media.

	Aqueous Medium			Non-Aqueous Medium		
Deconvoluted Peak	Slope (mV/dec)	*αn*	*k_app_* (s^−1^)	Slope (mV/dec)	*αn*	*k_app_* (s^−1^)
*i*	123	0.48	0.93	106	0.59	1.15
*ii*	131	0.45	0.88	135	0.44	0.86
*iii*	121	0.49	0.95	134	0.44	0.86

**Table 2:** Percent atomic ratios from XP spectra ^a^ of relevant functional groups in GO and erGO samples from aqueous and non-aqueous media.

Sample	C–OH	C–O–C	C=O	C=C_gr_^b^	C=C	COOH
**Aqueous medium**						
**GO_aq_**	8.8	17.7	16.8	-	54.5	2.1
**erGO_aq_**	25.8	2.8	6.1	32.8	24.4	3.6
**Non-aqueous medium**						
**GO_non-aq_**	7.0	24.6	10.8	-	54.9	2.7
**erGO_non-aq_**	8.9	4.0	2.1	45.3	38.8	0.7

^a^ Ratios are determined from the area of single components divided by the overall C 1s signal. Associated error is ±10%. ^b^ Component at 284.46 eV in erGO samples.

**Table 3:** Relevant parameters of Raman spectra as resulting from curve-fitting: position and full-width-at-half-maximum (FWHM) values of D, G, D’, 2D and D + G peaks; *I_D_/I_G_* and L_a_ values; and number of layers.

	D	G	D’	2D	D + G	*I_D_*/*I_G_*	L_a_ (nm) ^a^	# layers ^b^
	Position (cm^−1^)/FWHM (cm^−1^)							
**GO**	1355/ 118	1606/ 86	-	2708/ 194	2941/ 169	0.73	19	11
**erGO_aq_**	1365/ 74	1601/ 51	1630/ 34	2699/ 143	2953/ 131	1.12	12	8
**erGO_non-aq_**	1355/ 66	1592/ 45	1622/ 30	2704/ 137	2954/ 121	1.27	11	7

^a^ According to [55] ^b^ According to [52].

## Data Availability

Not applicable.

## Supplementary Materials

The following are available online at https://www.mdpi.com/article/10.3390/nano12010043/s1, Figures S1–S3: Deconvolution of cathodic waves related to the electrochemical reduction of a GO-coated silicon electrode in 0.1 M TBAPF_6_/CH_3_CN solution at 5, 10 and 50 mVs^−1^ potential scan rates, Figure S4: C 1s XP spectrum of GO_non-aq_ sample, Figures S5 and S6: Wide XPS spectra of erGO samples, Table S1: Numerical values of Figure 6.
