# Supplementary material for: Effect of Electrolytic Medium on the Electrochemical Reduction of Graphene Oxide on Si(111) as Probed by XPS
Source: Nanomaterials (Basel). 2021 Dec 23;12(1):43. doi: 10.3390/nano12010043 (PMC8747037; doi:10.3390/nano12010043)
Supplement: Supplementary file 1 [file nanomaterials-12-00043-s001.zip › nanomaterials-1503327-supplementary.pdf]

nanomaterials

MDPI

supplementary materials

Effect of Electrolytic Medium on the Electrochemical Reduction of Graphene Oxide on Si(111) as Probed by XPS

Andrea G. Marrani [1,*], Alessandro Motta [2], Francesco Amato [1], Ricardo Schrebler [3], Robertino Zanoni [1,*] and Enrique A. Dalchiele [4]

[1] Dipartimento di Chimica, Università di Roma La Sapienza, p.le A. Moro 5, I-00185 Rome, Italy; francesco.amato@uniroma1.it
[2] Dipartimento di Chimica, Università di Roma La Sapienza and Instm UdR, Roma p.le A. Moro 5, I-00185 Rome, Italy; alessandro.motta@uniroma1.it
[3] Instituto de Química, Facultad de Ciencias, Pontificia Universidad Católica de Valparaíso, Av. Brasil, 2950 Valparaiso, Chile; ricardo.schrebler@pucv.cl
[4] Instituto de Física, Facultad de Ingeniería, Universidad de la República, Julio Herrera y Reissig 565, C.C. 30, 11000 Montevideo, Uruguay; dalchiel@fing.edu.uy
* Correspondence: andrea.marrani@uniroma1.it (A.G.M.); robertino.zanoni@uniroma1.it (R.Z.); Tel.: +39-06-4991-3316 (A.G.M.); +39-06-4991-3328 (R.Z.)

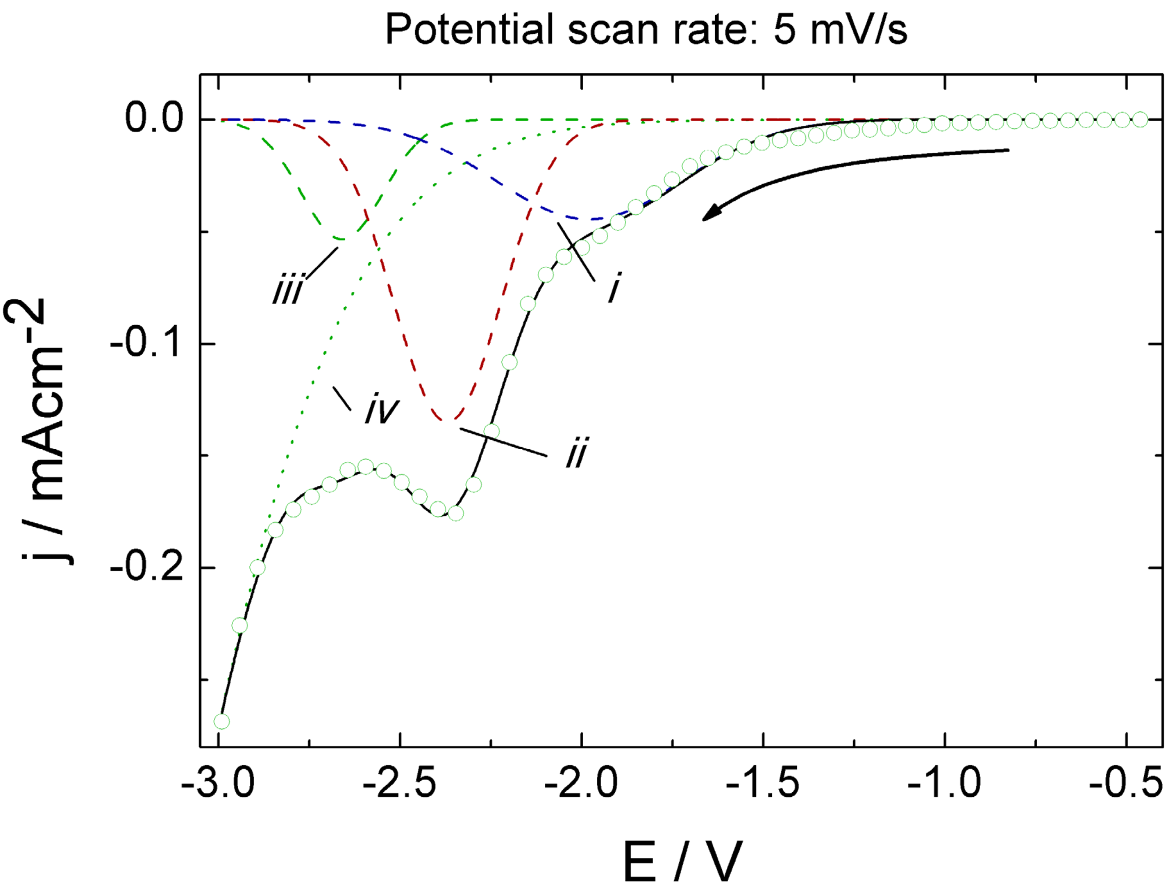

Figure S1. Deconvolution of cathodic wave related to the electrochemical reduction of a GO-coated silicon electrode in 0.1 M $TBAPF_6/CH_3CN$ solution at 5 mVs^{-1} potential scan rate. The graph shows: experimental current-potential data (olive open circles), deconvolution peaks (coloured dashed lines, from *i* to *iii* as indicated) and simulated envelope voltammogram (black solid line). The solvent contribution curve *iv* is also depicted. The reference electrode was a silver wire immersed in 0.01 M $AgNO_3$/0.1 M tetrabutylammonium perchlorate (TBAP, Sigma-Aldrich) in CH_3CN (+0.544 V vs. NHE, +0.345 V vs. Ag/AgCl).

i

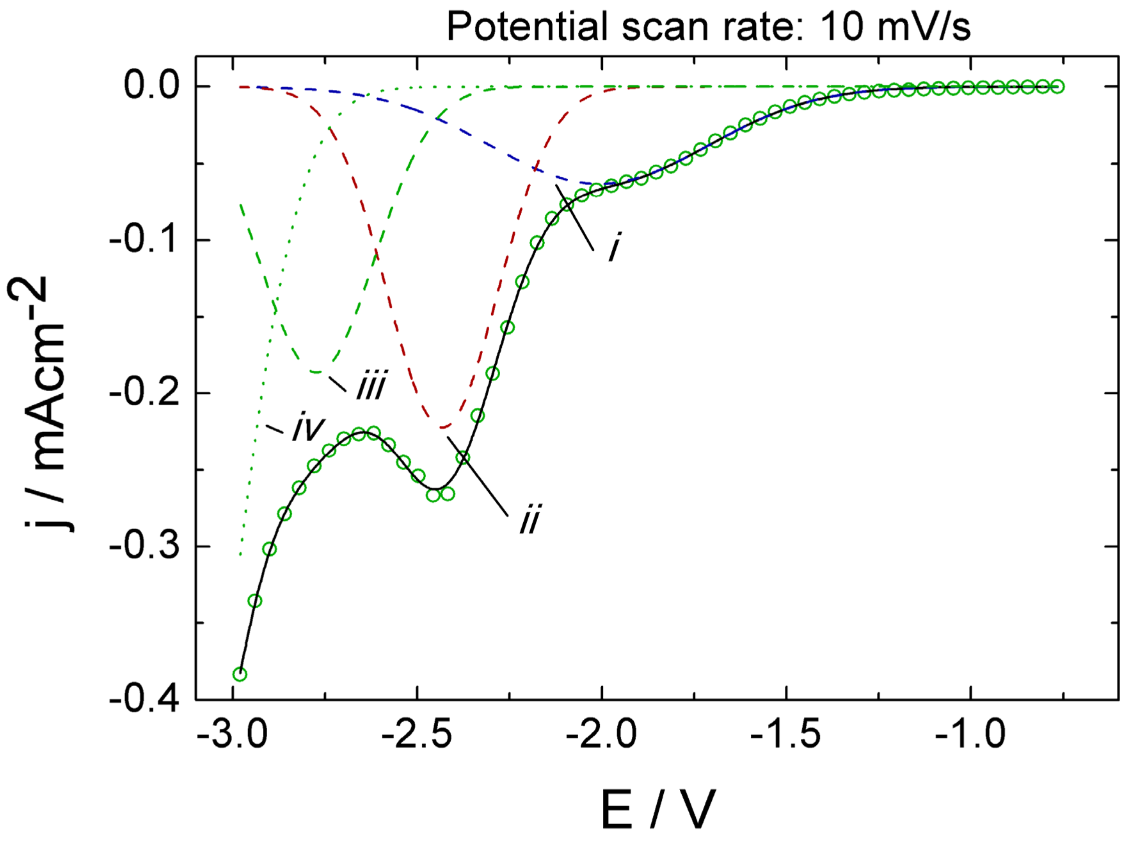

Figure S2. Deconvolution of cathodic wave related to the electrochemical reduction of a GO-coated silicon electrode in 0.1 M $TBAPF_6/CH_3CN$ solution at 10 mVs^{-1} potential scan rate. The graph shows: experimental current-potential data (olive open circles), deconvolution peaks (coloured dashed lines, from *i* to *iii* as indicated) and simulated envelope voltammogram (black solid line). The solvent contribution curve *iv* is also depicted. The reference electrode was a silver wire immersed in 0.01 M $AgNO_3$/0.1 M tetrabutylammonium perchlorate (TBAP, Sigma-Aldrich) in CH_3CN (+0.544 V vs. NHE, +0.345 V vs. Ag/AgCl).

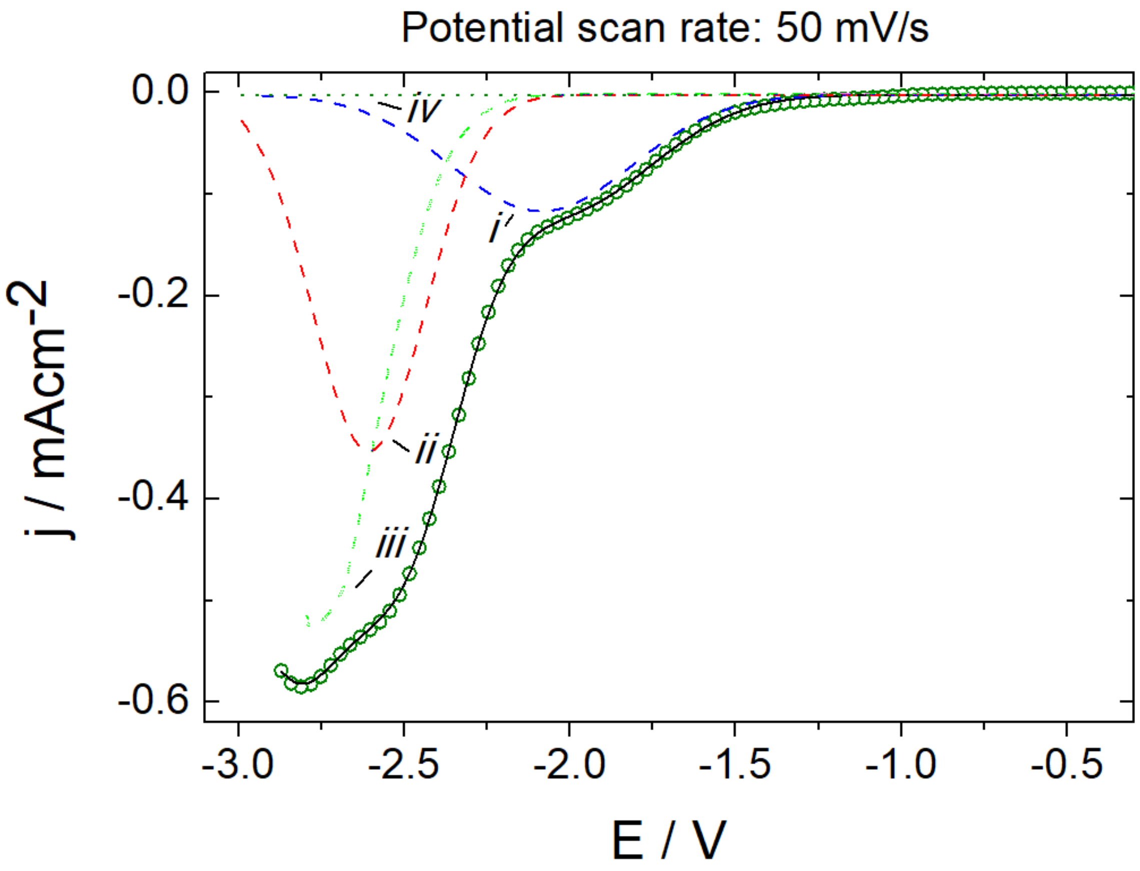

Figure S3. Deconvolution of cathodic wave related to the electrochemical reduction of a GO-coated silicon electrode in 0.1 M $TBAPF_6/CH_3CN$ solution at 50 mVs^{-1} potential scan rate. The graph shows: experimental current-potential data (olive open circles), deconvolution peaks (coloured dashed lines, from *i* to *iii* as indicated) and simulated envelope voltammogram (black solid line). The solvent contribution curve *iv* is also depicted. The reference electrode was a silver wire immersed in 0.01 M $AgNO_3$/0.1 M tetrabutylammonium perchlorate (TBAP, Sigma-Aldrich) in CH_3CN (+0.544 V vs. NHE, +0.345 V vs. Ag/AgCl).

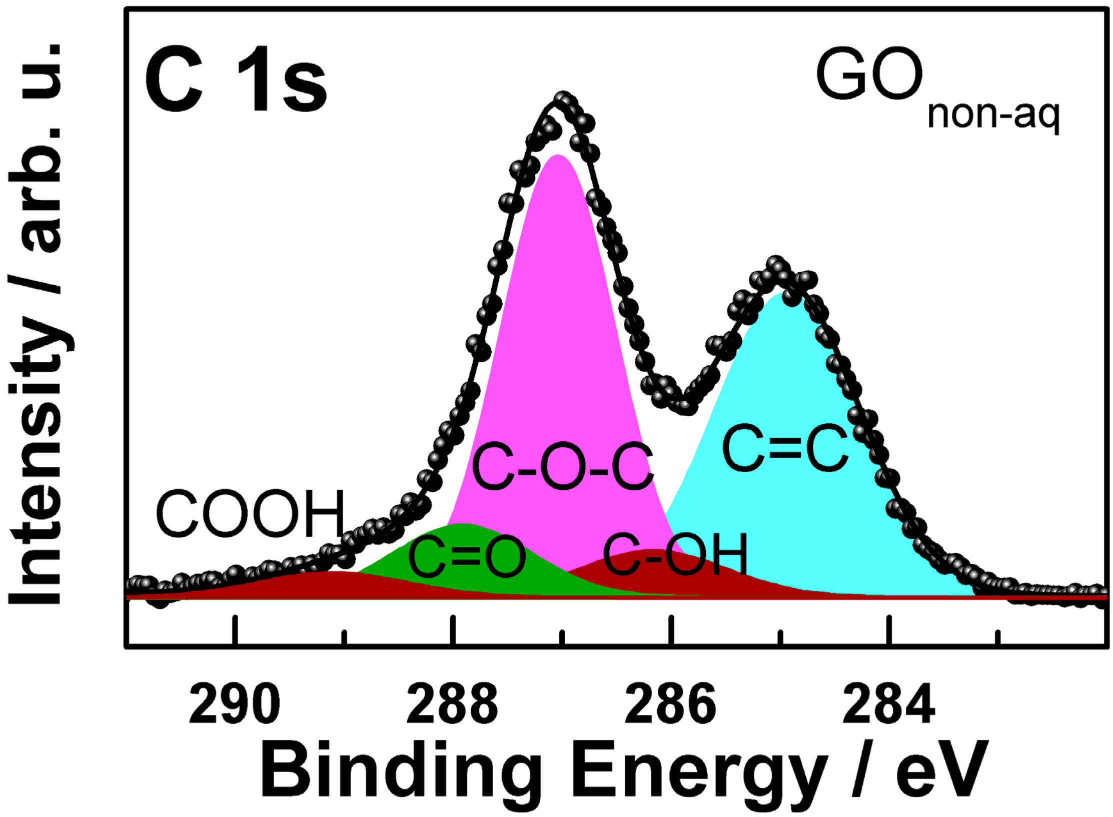

Figure S4. C 1s XP spectrum of pristine GO_{non-aq} sample. Raw data are displayed with dots, while fitting reconstruction with a continuous line and color filled curves.

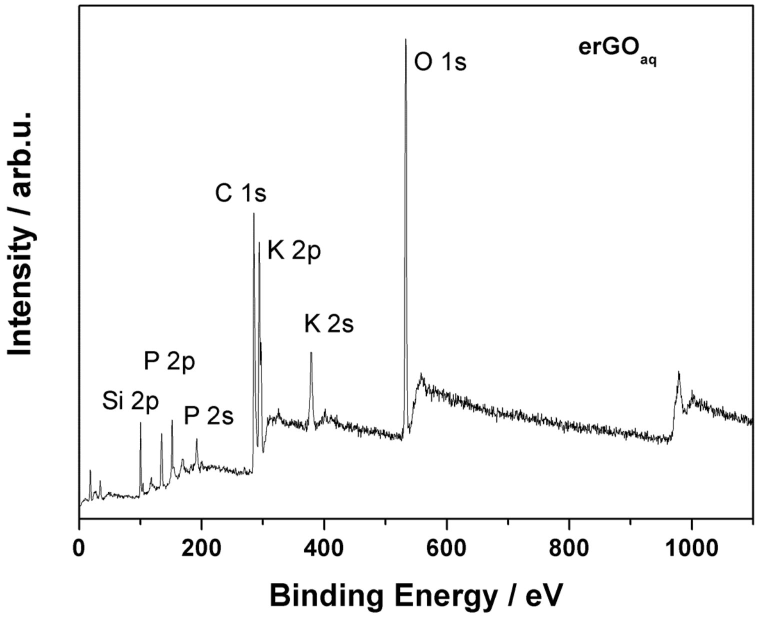

Figure S5. Wide XP spectrum of $erGO_{aq}$ sample.

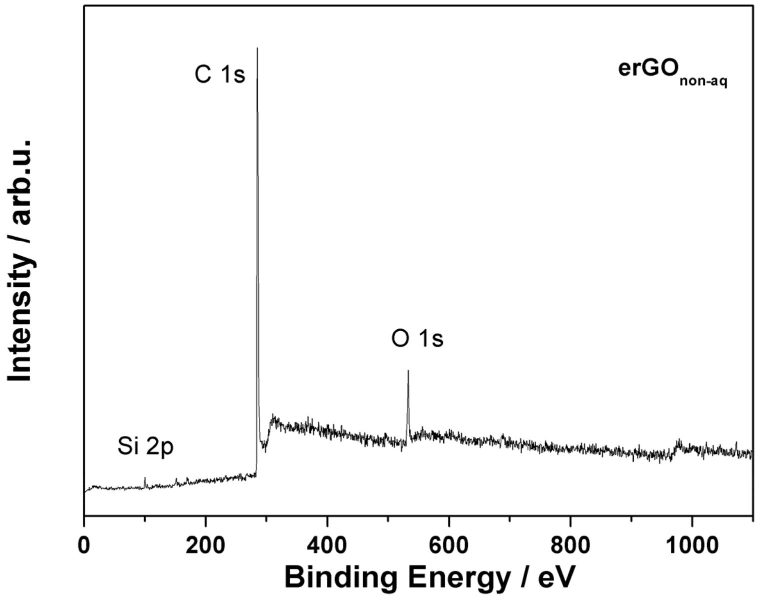

Figure S6. Wide XP spectrum of erGO$_{non-aq}$ sample.

Table S1. Relative removal degree percentage of four different OFGs (as indicated) after the electrochemical reduction process of typical GO-coated Si electrodes in and aqueous medium (1.0 M pH=7.2 PBS buffer solution) and in a non-aqueous one (0.1 M $TBAPF_6/CH_3CN$ solution). The potential scan rate was 20 mV s^{-1} and for a whole electrochemical process of five cycles. In all cases these OFG content values were calculated from the C 1s XPS spectra.

Sample	C–OH	C–O–C	C=O	COOH
**erGO$_{aq}$**	−193.0	84.2	63.7	−71.0
**erGO$_{non-aq}$**	−27.0	83.7	80.6	74.1

Oxygen/carbon atomic ratio ($R_{O/C}$) calculation from XPS spectra

The oxygen-to-carbon ratio ($R_{O/C}$) was calculated from the XPS data. Due to the inevitable absorption of water molecules and oxidation of H-Si, the O 1s peak includes these components besides the oxygen in GO, and hence it is not appropriate to directly use O 1s peak area to calculate $R_{O/C}$. Then, as proposed by Yudi Tu et al. [1,2], a semiquantitative approach has been followed. The percentages of different oxygenated groups (P_X, X refers to the different surface-bound oxygenated groups of GO) in C 1s spectra are used to calculate $R_{O/C}$ through the following equation S1:

$$R_{O/C} = \frac{P_{C-OH} + 1/2 P_{Epoxide} + P_{C=O} + 2P_{COOH}}{P_{C=C} + P_{C-OH} + P_{Epoxide} + P_{C=O} + P_{COOH}} \quad \text{(S1)}$$

References

1. Tu, Y.; Utsunomiya, T.; Kokufu, S.; Soga, M.; Ichii, T.; Sugimura, H. Immobilization of Reduced Graphene Oxide on Hydrogen-Terminated Silicon Substrate as a Transparent Conductive Protector, *Langmuir* **2017**, *33*, 10765–10771.
2. Tu, Y.; Nakamoto, H.; Ichii, T.; Utsunomiya, T.; Khatri, O.P.; Sugimura, H. Fabrication of reduced graphene oxide micro patterns by vacuum-ultraviolet irradiation: From chemical and structural evolution to improving patterning precision by light collimation, *Carbon* **2017**, *119*, 82–90.
